# The contribution of common genetic risk variants for ADHD to a general factor of childhood psychopathology

**DOI:** 10.1038/s41380-018-0109-2

**Published:** 2018-06-22

**Authors:** Isabell Brikell, Henrik Larsson, Yi Lu, Erik Pettersson, Qi Chen, Ralf Kuja-Halkola, Robert Karlsson, Benjamin B Lahey, Paul Lichtenstein, Joanna Martin

**Affiliations:** 1 Department of Medical Epidemiology and Biostatistics, Karolinska Institutet, Stockholm, Sweden; 2 School of Medical Sciences, Örebro University, Örebro, Sweden; 3 Statistical Genetics, Genetics and Computational Biology Department, QIMR Berghofer Medical Research Institute, Brisbane, QLD Australia; 4 Department of Public Health Sciences, University of Chicago, Chicago, IL USA; 5 MRC Centre for Neuropsychiatric Genetics and Genomics, Cardiff University, Cardiff, UK

**Keywords:** ADHD, Genetics

## Abstract

Common genetic risk variants have been implicated in the etiology of clinical attention-deficit/hyperactivity disorder (ADHD) diagnoses and symptoms in the general population. However, given the extensive comorbidity across ADHD and other psychiatric conditions, the extent to which genetic variants associated with ADHD also influence broader psychopathology dimensions remains unclear. The aim of this study was to evaluate the associations between ADHD polygenic risk scores (PRS) and a broad range of childhood psychiatric symptoms, and to quantify the extent to which such associations can be attributed to a general factor of childhood psychopathology. We derived ADHD PRS for 13,457 children aged 9 or 12 from the Child and Adolescent Twin Study in Sweden, using results from an independent meta-analysis of genome-wide association studies of ADHD diagnosis and symptoms. We estimated associations between ADHD PRS, a general psychopathology factor, and several dimensions of neurodevelopmental, externalizing, and internalizing symptoms, using structural equation modeling. Higher ADHD PRS were statistically significantly associated with elevated neurodevelopmental, externalizing, and depressive symptoms (*R*^*2*^ = 0.26–1.69%), but not with anxiety. After accounting for a general psychopathology factor, on which all symptoms loaded positively (mean loading = 0.50, range = 0.09–0.91), an association with specific hyperactivity/impulsivity remained significant. ADHD PRS explained ~ 1% (*p* value < 0.0001) of the variance in the general psychopathology factor and ~ 0.50% (*p* value < 0.0001) in specific hyperactivity/impulsivity. Our results suggest that common genetic risk variants associated with ADHD, and captured by PRS, also influence a general genetic liability towards broad childhood psychopathology in the general population, in addition to a specific association with hyperactivity/impulsivity symptoms.

## Introduction

Attention-deficit/hyperactivity disorder (ADHD) is a common neurodevelopmental disorder that affects 5–10% of children and is characterized by excessive inattentive, hyperactive, and impulsive symptoms [1]. It is well-established that genetic factors contribute to ADHD liability; twin and family studies consistently estimate the heritability of ADHD at 70–80% [2–7]. More recently, the largest genome-wide association study (GWAS) of clinical ADHD to date identified the first genome-wide significant loci associated with ADHD, and estimated the proportion of phenotypic variance explained by measured single nucleotide polymorphisms (SNPs) at 22% (standard error (SE) = 0.01) [8]. Significant SNP-heritability has also been reported by the largest GWAS of ADHD symptoms in population-based samples (5%(SE = 0.06) to 34%(SE = 0.17)) [9], with a near-complete sharing of genetic risks reported across these different ADHD definitions (*r*_g_ = 0.94(SE = 0.20)) [8].

In addition to evidence of a strong genetic liability, it is well known that ADHD is highly comorbid with other psychiatric conditions [10]. One important question is therefore the degree to which genetic risk implicated in ADHD is disorder-specific. Findings from population-based twin studies have reported moderate to strong genetic correlations between ADHD and other childhood psychiatric conditions, including autistic traits (*r*_g_ range 0.54–87) [11], learning disabilities (*r*_g_ range 0.31–41) [12–14], oppositional defiant and conduct problems (*r*_g_ range 0.46–74) [15–17], anxiety (*r*_g_ range 0.45–58), and depression (*r*_g_ range 0.34–77) [11]. Molecular genetic studies also support a genetic overlap between ADHD and a broad range of psychiatric conditions. For example, ADHD polygenic risk scores (i.e., weighted sum scores of an individual’s estimated total burden of risk alleles associated with a phenotype (PRS)) [18] have been associated with lower educational attainment, cognition, and conduct problems [8, 19–22]. Mixed findings have been reported for ADHD PRS and associations with autism and depression [23–28].

Based on the extensive phenotypic and genetic overlap among psychiatric disorders, it has been suggested that comorbidity may be attributed to a general factor of psychopathology that increases risk for virtually all prevalent psychiatric conditions [29, 30]. Twin and sibling studies have shown that a single latent shared genetic factor can account for on average 45% of variance in childhood externalizing, internalizing, and phobia symptoms [29, 31], 31% of variance in childhood neurodevelopmental symptoms [32], and 22% of disorder liability in several clinical psychiatric diagnoses, including ADHD [33]. Further, the twin-based heritability of a latent general psychopathology factor has been estimated at 43% in one study [31] and the SNP-heritability to 18%(SE = 0.10) [34] and 38%(SE = 0.16) [35] in two separate population-based pediatric samples.

These findings suggest that the co-occurrence of childhood psychiatric conditions is, at least in part, due to shared common genetic risk variants. Given the extensive comorbidity in ADHD and shared genetic risks with other psychopathology, it can thus be hypothesized that a proportion of the genetic risk variants associated with ADHD in recent GWAS [8] might not be disorder-specific, but rather act to increase risk for general childhood psychopathology more broadly. However, we are not aware of any studies addressing this question using molecular genetic data. The aims of the current study were therefore to: (1) examine whether ADHD PRS are associated with a range of neurodevelopmental, externalizing, and internalizing symptom dimensions in a large general population sample, and (2) quantify the extent to which any observed associations between ADHD PRS and the aforementioned symptom dimensions can be attributed to a general childhood psychopathology factor.

## Methods

### Study population

The Child and Adolescent Twin Study in Sweden (CATSS) is an ongoing study targeting all 9-year-old (born after June 1995) and 12-year-old (born before July 1995) twins born in Sweden since July 1992. Parents were contacted for a telephone interview on the twins’ 9th or 12th birthdays. The overall response rate in CATSS is 80% [36]. Analyses comparing non-responders and responders have shown that participating families generally have higher socio-economic status, lower rates of parental psychiatric illness, and child clinical diagnosis for ADHD, autism spectrum disorder (ASD), and learning disabilities [36]. CATSS was approved by the ethics committee at Karolinska Institutet and all participants gave informed consent. The study has been described in detail elsewhere [36].

### Genotyping and imputation

A total of 11,551 CATSS twins were genotyped using the Illumina Infinium PsychArray-24 BeadChip. Prior to analysis, stringent quality control (QC) procedures were performed on the genotyped markers and individuals using standardized procedures. After QC, 561,187 genotyped SNPs and 11,081 samples were retained. Genotypes for another 2495 monozygotic (MZ) twins were imputed from their genotyped co-twin, resulting in a sample size of 13,576 samples with genotype data. Details of the QC protocol, imputation, and principal components extraction are presented in supplementary note 1 and Figure S1. CATSS participants without available genetic data differed to those included in the current sample, in that they showed higher levels of parent-reported ADHD symptoms and clinical ADHD diagnosis were more likely to be male, and have parents with lower education levels (see supplementary Table S1).

### Polygenic risk scores

ADHD polygenic risk scores (PRS) were generated in CATSS based on summary statistics from what should theoretically be the most powerful discovery sample available : a meta-analysis of the largest GWAS of clinically diagnosed ADHD (20,183 cases, 35,191 controls) [8] and the largest GWAS of ADHD symptoms (17,666 children from population-based samples). Details of the discovery sample are provided in supplementary note 2 [9]. We calculated standardized betas for each SNP, based on available *z* scores, effective sample size and allele frequency in the discovery GWAS [37]. After excluding individuals with parent-reported cerebral palsy, Down syndrome, brain injury, and chromosomal abnormalities (supplementary Figure S1), ADHD PRS were derived in CATSS from best-guess imputed genotypes across a range of seven *p* value thresholds (0.00001 ≤ *P*_*T*_ ≤ 1). Indels, multi-allelic and symmetric/ambiguous SNPs were excluded. Autosomal SNPs with a minor allele frequency (MAF) ≥ 0.05 and good imputation quality (INFO score) ≥ 0.8 were clumped (linkage disequilibrium threshold *R*^2^ > 0.1, ± 1000 kb) using PLINK.v.1.9 [38]. Retained reference alleles were scored across the set of SNPs in PLINK (applying the command–score no-mean-imputation) using standard procedures [39, 40]. In line with previous publications, we used the PRS including SNPs at a threshold of *P*_*T*_ ≤ 0.50 for the main analysis [20, 41]. Sensitivity analyses of PRS associations across other *p* value thresholds are presented in the supplementary materials.

### Childhood psychiatric symptoms

Childhood psychiatric symptoms were assessed using the Autism-Tics, ADHD, and Other Comorbidities inventory (A-TAC). A-TAC is a 96-items questionnaire corresponding to DSM-IV definitions of childhood psychiatric disorders. Questions assess lifetime symptoms in relation to same-age peers [42]. We selected the 62 symptoms items measuring inattention (IA), hyperactivity/impulsivity (H/I), ASD, learning difficulties (LD), oppositional defiant disorder (ODD), conduct disorder (CD), depression (DEP) and anxiety (ANX). The A-TAC neurodevelopmental and externalizing scales have been validated, showing strong internal consistency and moderate to strong predictive validity [42–45]. A-TAC anxiety and depression items have not been validated and were only assessed in twins born from 1992 to 1997. For twins born after 1997, depression symptoms were instead assessed using the Short Mood and Feelings Questionnaire (SMFQ), a 13-item questionnaire measuring child depressive symptoms experienced in the last 2 weeks [46]. Anxiety symptoms were assessed using the Screen for Child Anxiety Related Emotional Disorders (SCARED), a 41-item questionnaire measuring symptoms experienced in the last three months across five anxiety dimensions: panic disorder (PD), generalized anxiety disorder (GAD), separation anxiety disorder (SAD), school anxiety (SA) and social phobia (SP) [47]. SMFQ and SCARED are validated questionnaires, with strong internal consistency and moderate predictive validity for clinical diagnoses [47–51].

All scales were rated according to three response categories: “no” (coded 0), “yes, to some extent” (coded 1), and “yes” (coded 2). As the A-TAC internalizing scales, SCARED, and SMFQ are not directly comparable measures, we used a split-sample approach based on the available internalizing assessments. The final sample sizes with genotype and phenotype data were 6603 (3483 unrelated individuals) for the A-TAC subsample, and 6854 (3634 unrelated individuals) in the SMFQ/SCARED subsample. Based on previous simulation studies, this sample size is more than adequate for estimating complex structural equation models (SEM) and PRS associations [52, 53].

### Statistical analyses

**Fig. 1:**
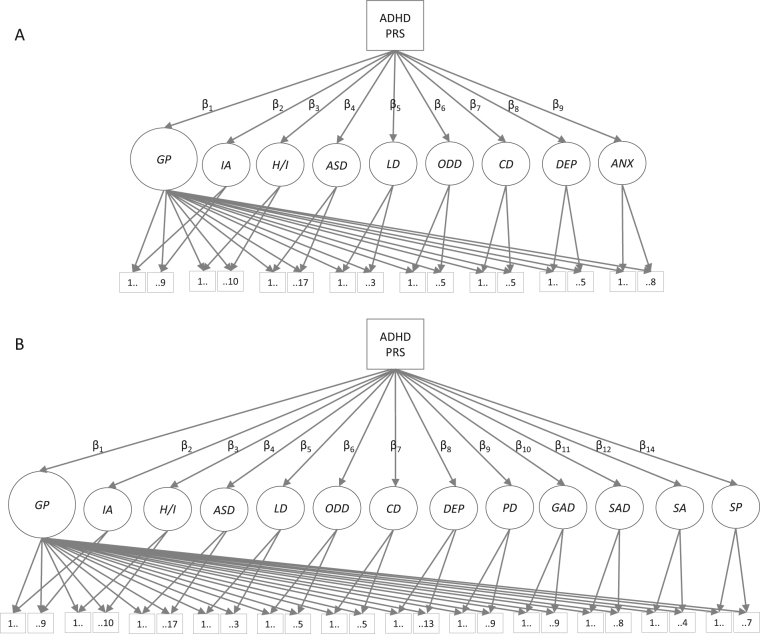
Path diagram of the general factor model in the A-TAC subsample **A** and the SMFQ/SCARED subsample **B**. Path diagram for the general factor models, presented by study subsample. Latent factors are depicted as circles. For clarity, covariates (age, sex and the six principal components) and correlations across latent trait factors are omitted in the above graphical representation. The models consisted of a latent general psychopathology factor (GP) and specific latent trait factors reflecting symptoms dimensions of inattention (IA), hyperactivity/impulsivity (H/I), autism spectrum disorder (ASD), learning difficulties (LD), oppositional defiant disorder (ODD), conduct disorder (CD), depression (DEP), and anxiety (ANX) or panic disorder (PD), generalized anxiety (GAD), separation anxiety (SAD), school anxiety (SA), and social phobia (SP). Variances for all latent factors were fixed at 1. Measured variables are depicted as squares, and include the ADHD PRS and all symptoms items from the Autism-Tics, ADHD, and Other Comorbidities inventory (A-TAC), the Short Mood and Feelings Questionnaire (SMFQ), and the Screen for Child Anxiety Related Emotional Disorders (SCARED). Numbers 1…X indicate the number of symptom items loading onto each specific latent trait factor. β_1_–β_x_ represent the regression coefficients, regressing each latent variable onto ADHD PRS. Note that the corresponding path diagrams for the correlated factors models are identical, excluding the general psychopathology factor

We estimated associations between ADHD PRS and ADHD symptom dimensions (IA, H/I) and related neurodevelopmental (ASD, LD), externalizing (ODD, CD), and internalizing (DEP, ANX) symptom dimensions, using confirmatory factor analysis and regression analyses implemented via SEM. Path diagrams of the models are presented by subsample in Fig. 1.

We first fitted a correlated factors model where symptoms from each subscale were set to load onto a corresponding single latent trait factor. All the latent trait factors were allowed to correlate. In the A-TAC subsample, a correlated factors model with eight latent trait factors was fitted, corresponding to symptoms dimensions of IA, H/I, ASD, LD, ODD, CD, DEP, and ANX. In the SMFQ/SCARED subsample, we fitted a correlated factors model with 12 latent trait factors, including the first six factors outlined above, one DEP factor measured via SMFQ, and five latent anxiety factors corresponding to the SCARED subscales of PD, GAD, SAD, SA, and SP. Previous studies of the SCARED have shown that this five-factor structure has the best psychometric properties for the questionaire [54–56].

Second, we fitted a general factor (or bifactor) model, which in addition to the latent trait factors, included a general psychopathology factor. The general factor model quantifies the extent to which covariance among symptom dimensions reflects both a general factor (on which all assessed symptoms load), and a number of specific latent trait factors (on which only a subset of the symptoms load) [57, 58]. Correlations between the specific latent trait factors and the general factor are fixed at zero, whereas correlations between the specific latent trait factors are free to vary.

In both models, the latent factors were regressed on ADHD PRS using SEM, with sex, age and the first six PCs (to account for population stratification) included as covariates. We evaluated whether the models provided a good fit to the underlying data using the comparative fit index, and the root mean square error of approximation (RMSEA) [59]. A likelihood ratio test was used to test whether the exclusion of the general psychopathology factor from the correlated factors model led to a significant decrease in model fit. To account for the non-independence of twin data, family clusters were specified and standard errors were estimated using a sandwich estimator. All models were run using Mplus [60].

### Sensitivity analyses

We performed several sensitivity analyses to test the robustness of results from the general factor model (see supplementary note 3 for details). First, to test whether observed associations were driven by ADHD cases, we re-ran analyses excluding children with an ADHD diagnosis (ICD and/or ≥ 8 A-TAC DSM-based symptoms). For completeness, we present the number children with an ICD diagnosis corresponding to the any of the assessed symptoms dimensions in supplementary Table S2. Second, we excluded one twin in every monozygotic pair to confirm that estimates were not inflated by the inclusion of genetically identical individuals. Third, we tested for sex differences in the association between ADHD PRS and the latent factors. Finally, we tested whether ADHD PRS showed similar associations with the latent factors across a range of seven *p* value thresholds (0.00001 ≤ *P*^T^ ≤ 1).

### Code availability

Computer codes are available upon request from the corresponding author.

## Results

### Correlated factors model

The correlated factors model fit the data well in both subsamples (CFI > 0.94 and RMSEA < 0.02) [59] (Table 1). All symptoms loaded positively and significantly onto their corresponding latent trait factor. Standardized factor loadings are reported in Table 2. Neurodevelopmental, externalizing, and internalizing latent factors were moderately to strongly correlated in the A-TAC subsample (mean *r* = 0.64, range = 0.44–83). Similar correlations were found in the SMFQ/SCARED subsample across neurodevelopmental, externalizing and the depression latent factors (mean *r* = 0.62, range = 0.40–84), whereas correlations with the latent anxiety factors were generally weaker (mean *r* = 0.40, range = 0.11–65). Notably, SP showed a particularly weak correlation with H/I (*r* = 0.11), and overall lower correlations with all latent factors (mean *r* = 0.33, range = 0.11–52). Correlations across the latent trait factors, before and after regression of PRS and covariates, are presented in supplementary Figures S2–S3.

**Table 1:** Model fit for the correlated factors model and the general factor model in the A-TAC subsample and the SMFQ/SCARED subsample

Model	CFI	RMSEA (90%CI)	*χ*^2^ (df)	Δ*χ*^2^	diff df	*p*
A-TAC subsample						
General factor model	0.97	0.02 (0.02–0.02)	6674.06 (2216)	–	–	–
Correlated factors model	0.95	0.02 (0.02–0.02)	8636.54 (2287)	1962.48	71	< .0001
SMFQ/SCARED subsample						
General factor model	0.96	0.01 (0.01–0.01)	12544.05 (5275)	–	–	–
Correlated factors model	0.94	0.02 (0.02–0.02)	16123.77 (5382)	3579.72	107	< .0001

*CFI*, comparative fit index. *RMSEA*, root mean square error of approximation. *χ*^2^, chi-square. *df*, degrees of freedom. The likelihood ratio (chi-square) test of model fit was run using the DIFFTEST option in MPlus, comparing the fit of the nested correlated factors model to the general factor model

**Table 2:** Standardized factor loadings from the correlated factors model and the general factor model in the A-TAC subsample and the SMFQ/SCARED subsample

Scale	Item	Latent trait factor loadings				GP loadings	
		Mod1	Mod2	Mod3	Mod4	Mod2	Mod4
A-TAC inattention	Fails to pay close attention to details?	0.80	0.20	0.77	0.25	0.78	0.73
	Difficulty sustaining attention in tasks?	0.91	0.02†	0.89	0.17	0.90	0.86
	Often does not listen when spoken to directly?	0.86	0.19	0.84	0.30	0.85	0.79
	Difficulty following instructions and finishing tasks?	0.91	-0.13	0.91	0.02†	0.91	0.91
	Difficulty organizing tasks/activities?	0.91	-0.09	0.92	0.06*	0.91	0.91
	Often avoid tasks that require sustained mental effort?	0.84	0.01†	0.80	0.02†	0.83	0.80
	Often loses things?	0.77	0.61	0.78	0.69	0.69	0.62
	Easily distracted/disturbed?	0.89	0.07*	0.87	0.20	0.89	0.84
	Often forgetful in daily activities?	0.79	0.57	0.79	0.64	0.70	0.63
A-TAC hyperactivity/impulsivity	Difficulties holding hands and feet still, cannot stay seated?	0.81	0.49	0.79	0.43	0.61	0.64
	Often move about in school or in other situations when s/he is supposed to remain seated?	0.84	0.40	0.82	0.37	0.68	0.68
	Often run around and climbs more than his/hers peers?	0.78	0.51	0.73	0.47	0.58	0.56
	Difficulty playing calmly and quietly?	0.88	0.53	0.89	0.45	0.68	0.72
	Often on the go or act as if driven by a motor?	0.85	0.58	0.85	0.53	0.63	0.65
	Talks excessively?	0.69	0.59	0.65	0.56	0.45	0.43
	Often blurt out answers?	0.74	0.63	0.75	0.59	0.49	0.52
	Difficulty awaiting turns?	0.88	0.63	0.87	0.63	0.62	0.62
	Often interrupt or intrude on others?	0.87	0.66	0.85	0.64	0.60	0.61
	Easily bored?	0.80	0.37	0.79	0.34	0.66	0.66
A-TAC autism spectrum disorder	Language development delayed?	0.45	0.19	0.40	0.05†	0.39	0.40
	Difficulties sustaining a conversation?	0.75	0.34	0.78	0.39	0.64	0.66
	Like to repeat words and expressions, use of words in a way other people find strange?	0.74	0.44	0.68	0.35	0.59	0.57
	Difficulties with pretend play or imitates considerably less than other children?	0.59	0.45	0.64	0.38	0.42	0.51
	Talk in too high pitch or too quietly?	0.53	0.35	0.53	0.30	0.40	0.42
	Difficulties keeping “on track” when telling other people something?	0.80	0.25	0.75	0.10	0.72	0.73
	Difficulties expressing emotions and reactions with facial gestures, prosody, or body language?	0.80	0.56	0.84	0.45	0.59	0.69
	Exhibits considerable difficulties interacting with peers?	0.86	0.64	0.86	0.53	0.62	0.68
	Uninterested in sharing joy, interests, and activities with others?	0.81	0.58	0.70	0.48	0.60	0.53
	Can only be with other people on own terms?	0.78	0.58	0.74	0.46	0.56	0.58
	Difficulties behaving as expected by peers?	0.90	0.58	0.89	0.48	0.69	0.73
	Easily influenced by other people?	0.77	0.25	0.66	0.22	0.68	0.60
	Absorbed by interests in a repetitive or too intense way?	0.72	0.47	0.68	0.42	0.56	0.54
	Absorbed by routines in a way that produces problems?	0.76	0.59	0.78	0.67	0.54	0.52
	Ever engaged in strange hand movements or walking high on tiptoe when s/he was happy or upset?	0.60	0.44	0.52	0.31	0.45	0.41
	Gets absorbed by details?	0.78	0.60	0.77	0.62	0.56	0.53
	Dislikes changes in daily routines?	0.71	0.59	0.74	0.65	0.49	0.50
A-TAC learning difficulties	More difficulties than expected acquiring reading skills?	0.74	0.51	0.75	0.50	0.61	0.62
	Is learning slow and laborious?	0.97	0.51	0.96	0.50	0.79	0.77
	Difficulties with basic maths?	0.83	0.48	0.80	0.53	0.68	0.66
A-TAC oppositional defiant disorder	Ever been so angry that s/he cannot be reached?	0.71	0.52	0.69	0.53	0.48	0.45
	Often argue with adults?	0.84	0.64	0.77	0.62	0.56	0.49
	Often tease others by deliberately doing things that are perceived as provocative?	0.74	0.52	0.73	0.53	0.51	0.50
	Easily offended or disturbed by others?	0.86	0.64	0.84	0.63	0.57	0.56
	Easily teased?	0.84	0.67	0.81	0.61	0.55	0.53
A-TAC conduct disorder	Ever been deliberately physically cruel to anybody?	0.75	0.65	0.72	0.62	0.46	0.44
	Often start fights?	0.79	0.78	0.80	0.72	0.45	0.48
	Often lie or cheat?	0.80	0.48	0.78	0.50	0.56	0.54
	Steal things?	0.85	0.53	0.89	0.59	0.60	0.62
	Ever engaged in shoplifting?	0.46	0.29	0.48	0.36	0.32	0.32
A-TAC depression	Poor self-confidence?	0.81	0.61	NA	NA	0.53	NA
	Often complain about bellyaches, headaches, breathing difficulties or other bodily symptoms?	0.56	0.55	NA	NA	0.32	NA
	Recurrent periods of obvious irritability?	0.86	0.61	NA	NA	0.58	NA
	Self-confidence vary considerably across situations?	0.82	0.64	NA	NA	0.53	NA
	Sleeping problems?	0.62	0.53	NA	NA	0.38	NA
A-TAC anxiety	Difficulty functioning outside the family home?	1.00	0.66	NA	NA	0.61	NA
	Often voice fears that family members may die or get hurt?	0.61	0.52	NA	NA	0.32	NA
	Unreasonable fear of being alone?	0.68	0.63	NA	NA	0.34	NA
	Difficulty sleeping if a family member is not around?	0.63	0.65	NA	NA	0.29	NA
	Complain of recurring headaches, bellyaches, nauseas, or vomits after separation from loved ones?	0.63	0.73	NA	NA	0.24	NA
	Panic attacks?	0.67	0.63	NA	NA	0.33	NA
	Fear leaving the home alone, being in crowds/waiting in line/riding bus or train?	0.73	0.59	NA	NA	0.40	NA
	Particularly nervous or anxious?	0.81	0.69	NA	NA	0.44	NA
SMFQ depression	Felt miserable and unhappy?	NA	NA	0.72	0.74	NA	0.28
	Did not enjoy anything at all?	NA	NA	0.64	0.59	NA	0.30
	Felt so tired that s/he just sat around and did nothing?	NA	NA	0.55	0.44	NA	0.31
	Very restless?	NA	NA	0.78	0.34	NA	0.60
	Felt s/he is no good anymore?	NA	NA	0.84	0.81	NA	0.34
	Cried a lot?	NA	NA	0.74	0.76	NA	0.28
	Found it hard to think properly or concentrate?	NA	NA	0.90	0.44	NA	0.66
	Hated him/herself?	NA	NA	0.85	0.80	NA	0.36
	Felt s/he was a bad person?	NA	NA	0.86	0.83	NA	0.33
	Felt lonely?	NA	NA	0.77	0.69	NA	0.38
	Felt unloved?	NA	NA	0.85	0.78	NA	0.38
	Thought s/he could never be as good as other kids?	NA	NA	0.84	0.76	NA	0.40
	Thought s/he did everything wrong?	NA	NA	0.85	0.80	NA	0.37
SCARED panic disorder	When frightened, s/he has difficulties breathing.	NA	NA	0.73	0.72	NA	0.26
	When frightened, s/he feels like passing out.	NA	NA	0.67	0.71	NA	0.19
	When frightened, s/he feels like s/he is going crazy.	NA	NA	0.92	0.66	NA	0.51
	When frightened, s/he feels like things are not real.	NA	NA	0.75	0.65	NA	0.34
	When frightened, her/his heart beats fast.	NA	NA	0.56	0.51	NA	0.23
	Really frightened for no reason at all.	NA	NA	0.59	0.58	NA	0.21
	When frightened, s/he feels like throwing up.	NA	NA	0.88	0.83	NA	0.34
	When frightened, s/he feels dizzy.	NA	NA	0.67	0.66	NA	0.24
	S/he gets shaky.	NA	NA	0.71	0.75	NA	0.21
SCARED generalized anxiety disorder	Worries about other people liking him/her.	NA	NA	0.75	0.66	NA	0.35
	Worries about being as good as other kids.	NA	NA	0.77	0.72	NA	0.32
	Worries about what is going to happen in the future.	NA	NA	0.79	0.75	NA	0.32
	Worries about how well s/he does things.	NA	NA	0.82	0.76	NA	0.34
	Worries about things that have already happened.	NA	NA	0.76	0.63	NA	0.39
	My child is nervous.	NA	NA	0.89	0.68	NA	0.51
	Worries about things working out for him/her.	NA	NA	0.82	0.78	NA	0.33
	Told s/he worries too much.	NA	NA	0.78	0.74	NA	0.32
	My child is a worrier.	NA	NA	0.82	0.81	NA	0.30
SCARED separation anxiety	Scared sleeping away from home.	NA	NA	0.64	0.65	NA	0.18
	My child follows me wherever I go.	NA	NA	0.83	0.54	NA	0.46
	Worries about sleeping alone.	NA	NA	0.70	0.63	NA	0.26
	Nightmares something bad will happen to her/his parents.	NA	NA	0.78	0.78	NA	0.23
	Nightmares something bad will happen to him/her.	NA	NA	0.68	0.68	NA	0.21
	Afraid to be alone in the home.	NA	NA	0.60	0.58	NA	0.19
	Does not like to be away from family.	NA	NA	0.64	0.66	NA	0.17
	Worries that something bad might happen to parents.	NA	NA	0.83	0.80	NA	0.26
SCARED school anxiety	Headaches at school.	NA	NA	0.58	0.50	NA	0.29
	Stomachaches at school.	NA	NA	0.81	0.79	NA	0.34
	Worries about going to school.	NA	NA	0.95	0.84	NA	0.46
	Scared to go to school.	NA	NA	0.98	0.85	NA	0.49
SCARED social phobia	Does not like to be with people s/he does not know well.	NA	NA	0.82	0.77	NA	0.26
	Nervous with people s/he does not know well.	NA	NA	0.90	0.82	NA	0.33
	Difficulties talking to people s/he does not know well.	NA	NA	0.87	0.86	NA	0.23
	Shy with people s/he does not know well.	NA	NA	0.84	0.90	NA	0.09
	Nervous if s/he has do something, whereas others watch.	NA	NA	0.77	0.61	NA	0.37
	Nervous when s/he is going to a place where there will be people that s/he does not know well.	NA	NA	0.83	0.74	NA	0.32
	My child is shy.	NA	NA	0.81	0.85	NA	0.09

Mod1: correlated factors model in A-TAC subsample

Mod2: general factor model in A-TAC subsample

Mod3: correlated factors model in SMFQ/SCARED subsample

Mod4: general factor model in SMFQ/SCARED subsample

Note: GP loadings, loadings onto the general psychopathology factor for all items. Latent trait factor loadings, loadings onto each latent trait factor corresponding to the specific item scales of A-TAC, SMFQ, and SCARED. † Not significant at *p* ≤ 0.05; * Significant at *p* ≤ 0.001. All other estimates significant at *p* ≤ 0.0001. Four items from the PD scale had to be excluded from the model due very few endorsements, leading to estimation problems of the polychoric correlations. The items were “People tell to my child that s/he looks nervous”, “When my child gets frightened, s/he sweats a lot”, “When my child gets frightened, s/he feels like s/he is choking”, and “My child is afraid of having anxiety (or panic) attacks”

In both subsamples, higher ADHD PRS were statistically significantly associated with higher symptom levels in all latent neurodevelopment, externalizing, and depression factors, after adjusting for covariates. ADHD PRS was not statistically significantly associated with any of the latent anxiety factors, with the exception of the latent PD factor (*β* = 0.06, *p* = 0.011). Standardized regression results for PRS *p* value threshold ≤ 0.5 are reported in Table 3, and results across *p* value thresholds in supplementary Figures S4–S5.

**Table 3:** Association between ADHD PRS and latent trait factors in the correlated factors model and the general factor model (PRS *p* value threshold < 0.5)

	Correlated factors model				General factor model			
Latent factor	Beta	S.E	*p*	*R*^2^	Beta	S.E	*p*	*R*^2^
A-TAC subsample (*N* = 6603)								
GP	NA	NA	NA	NA	**0.09**	**0.02**	**<** **.0001**	**0.86%**
IA	**0.09**	**0.02**	**<** **0.0001**	**0.83%**	−0.01	0.02	0.929	0.00%
H/I	**0.11**	**0.02**	**<** **0.0001**	**1.19%**	**0.06**	**0.02**	**0.003**	**0.37%**
ASD	**0.07**	**0.02**	**<** **0.0001**	**0.50%**	−0.01	0.03	0.862	0.00%
LD	**0.07**	**0.02**	**<** **0.0001**	**0.53%**	−0.01	0.03	0.873	0.00%
ODD	**0.06**	**0.02**	**<** **0.001**	**0.41%**	0.01	0.02	0.895	0.00%
CD	**0.08**	**0.03**	**0.007**	**0.69%**	0.03	0.04	0.390	0.12%
DEP	**0.05**	**0.02**	**0.009**	**0.26%**	−0.01	0.02	0.564	0.01%
ANX	0.05	0.02	0.053	0.22%	0.00	0.03	0.998	0.00%
SCARED/SMFQ subsample (*N* = 6854)								
GP	NA	NA	NA	NA	**0.10**	**0.02**	**<0.0001**	**1.06%**
IA	**0.10**	**0.02**	**<0.0001**	**1.08%**	0.02	0.02	0.482	0.02%
H/I	**0.13**	**0.02**	**<0.0001**	**1.69%**	**0.08**	**0.02**	**<0.0001**	**0.69%**
ASD	**0.06**	**0.02**	**0.001**	**0.40%**	−0.03	0.02	0.220	0.08%
LD	**0.07**	**0.02**	**0.002**	**0.45%**	−0.03	0.03	0.308	0.08%
ODD	**0.10**	**0.02**	**<0.0001**	**0.98%**	0.04	0.02	0.058	0.17%
CD	**0.11**	**0.03**	**<.0001**	**1.19%**	0.05	0.03	0.117	0.26%
DEP	**0.07**	**0.02**	**<.001**	**0.42%**	0.02	0.02	0.411	0.03%
PD	**0.06**	**0.03**	**0.014**	**0.41%**	0.02	0.03	0.405	0.05%
GAD	0.03	0.02	0.066	0.10%	−0.01	0.02	0.450	0.02%
SAD	0.01	0.02	0.826	0.00%	−0.03	0.02	0.071	0.10%
SA	0.00	0.03	0.996	0.00%	−0.05	0.03	0.052	0.27%
SP	−0.02	0.02	0.272	0.04%	**−0.05**	**0.02**	**0.004**	**0.24%**

All models are adjusted for sex, age, and six principal components. Reported betas are standardized. S.E, standard error. *R*^2^, variance explained (beta^2^). Significant estimates are in bold. Results from the correlated factors model reflect associations between ADHD PRS prior to accounting for covariance across all symptoms via the general psychopathology factor. *GP*, general psychopathology. *IA*, inattention. *H/I*, hyperactivity/impulsivity. *ASD*, autism spectrum disorder. *LD*, learning difficulties. *ODD*, oppositional defiant disorder. *CD*, conduct disorder. *DEP*, depression. *ANX*, anxiety. *PD*, panic disorder. *GAD*, generalized anxiety disorder. *SAD*, separation anxiety disorder. *SA*, school anxiety. *SP*, social phobia

### General factor model

The general factor model also fit the data well in both subsamples (CFI > 0.96, RMSEA < 0.02). Furthermore, omitting the general psychopathology factor resulted in a statistically significant decrease in model fit (Table 1). Standardized factor loadings are presented in Table 2. In both subsamples, all symptoms loaded positively and significantly onto the general psychopathology factor. Mean loadings were strongest for neurodevelopmental symptoms (A-TAC subsample mean loading = 0.64, range = 0.39–91: SMFQ/SCARED subsample mean loading = 0.64, range = 0.40–91), slightly lower for externalizing symptoms (A-TAC subsample mean loading = 0.51, range = 0.32–60: SMFQ/SCARED subsample mean loading = 0.49, range = 0.32–62) and weakest for internalizing symptoms (A-TAC subsample mean loading = 0.41, range = 0.24–61: SMFQ/SCARED subsample mean loading = 0.32, range = 0.09–66). The general psychopathology factor explained 56% of the covariance across traits (explained common variance) in the A-TAC subsample and 40% in the SMFQ/SCARED subsample [61]. Correlations across the specific latent trait factors were attenuated in the general factor model. Notably, correlations between LD and all other factors were attenuated towards the null or showed an inverse association. Further, SP became significantly negatively correlated with IA and H/I (supplementary Figures S2–S3).

In both subsamples, higher ADHD PRS were significantly associated with the general psychopathology factor (*β* = 0.09–10, *p* < 0.0001), explaining ~ 1% of the variance in the general factor after adjusting for covariates. After accounting for covariance across all symptoms via the general psychopathology factor, the association between ADHD PRS and the specific latent H/I factor remained significant in both subsamples (*β* = 0.06–8, *p* < 0.0001), explaining 0.37–69% of the variance in the specific H/I factor after adjusting for covariates. In the SMFQ/SCARED subsample, we also observed a significant negative association between ADHD PRS and the specific latent SP factor (*β* = −0.05, *p* = 0.004). Standardized regression results for ADHD PRS *p* value threshold ≤ 0.5 are reported in Table 3, and across the range of *p* value thresholds in supplementary figures S4–S5.

### Sensitivity analyses

After excluding ADHD cases, ADHD PRS remained statistically significantly associated with the general psychopathology factor and the specific H/I factor in both subsamples. Results excluding one MZ twin per pair did not differ markedly from the main analyses. Analyses testing for sex differences in the association between ADHD PRS and the latent factors showed a similar pattern of results as the main analyses, although PRS associations were generally stronger in males (general factor *R*^2^ = 1.00–14%: H/I *R*^2^ = 0.81%), than in females (general factor *R*^2^ = 0.36–81%: H/I *R*^2^ = 0.03–1.00%).

## Discussion

Results from this study show that common GWAS variants that increase the risk for ADHD as captured by PRS, are not only associated with ADHD symptoms (*R*^2^ = 0.83–1.69%) in an independent population-based sample, but also with a range of childhood neurodevelopmental (*R*^2^ = 0.40–0.53%), externalizing (*R*^2^ = 0.41–1.19%), and to a lesser extent, internalizing symptom dimensions (*R*^2^ = 0–0.41%). Importantly, when modeling the shared variance across these symptom dimensions, we found that the associations were largely accounted for by a general childhood psychopathology factor. The significant association between ADHD PRS and a general psychopathology factor (*R*^2^ = 0.86–1.06%) suggests that a considerable portion of the genetic variants associated with ADHD, that are captured by PRS and shared with other measures, reflect a non-specific genetic liability toward broad childhood psychopathology.

Beyond the association between ADHD PRS and a general psychopathology factor, results also showed a unique association between ADHD PRS and specific H/I (*R*^2^ = 0.37–0.69%). About 2/3 of the association between ADHD PRS and H/I could be attributed to general variance shared across childhood psychopathology symptoms, and ~ 1/3 to variance specific to hyperactivity/impulsivity. These finding were robust across both subsamples and provide important molecular genetic confirmation of results from previous twin studies, showing that whilst a substantial proportion of genetic influences on ADHD symptoms are shared with a general psychopathology factor, there are also ADHD-specific genetic influences [29, 32, 33].

In contrast, there was no significant association between ADHD PRS and specific inattention after accounting for covariance across all symptom dimensions via the general factor. There are several potential reasons for the differential pattern of ADHD PRS association across ADHD symptom dimensions; it is possible that hyperactive/impulsive symptoms are stronger drivers of ADHD diagnosis, leading to an overrepresentation of combined and primarily hyperactive/impulsive ADHD cases in the clinical discovery GWAS. The lack of association between ADHD PRS and specific inattention is likely also explained by the fact that the majority of the inattentive symptoms loaded very strongly onto the general psychopathology factor, leaving little variance in the specific inattention factor. This may suggest that inattention is phenotypically and genetically more closely linked to a general liability for childhood psychopathology, or it may reflect measurement properties of the A-TAC. To disentangle these explanations, results would need to be replicated in a different sample using other measures and/or raters.

In the correlated factors model, we found significant ADHD PRS associations with neurodevelopmental, externalizing, and depression symptom dimensions, but not with anxiety. Anxiety symptoms also showed the weakest loadings onto a general factor, both when measured by A-TAC and SCARED. These results suggest that anxiety may be less genetically associated with ADHD in childhood, as compared with other psychopathology dimensions. Such a conclusion is generally consistent with findings from twin and molecular studies [11]. Nonetheless, it is also possible that parent ratings do not fully capture variation in child anxiety symptoms at this age [51], which may in part explain the observed overall weaker associations seen for anxiety. Somewhat surprisingly, we observed a significant negative association between ADHD PRS and specific SP in the general factor model in the SMFQ/SCARED subsample. SP was also the only latent anxiety factor to show a significant negative association with ADHD symptoms in the general factor model. This suggests that the negative association between ADHD PRS and SP may reflect the previously reported tendency for internalizing and externalizing symptom dimensions to become inversely associated in general factor models [30, 62, 63].

Analyses stratified by sex generally showed stronger PRS associations in males relative to females, particularly for associations with the general psychopathology factor and specific hyperactivity/impulsivity. Although this could reflect sex-specific genetic differences, it is more likely to be explained by lower levels of hyperactivity/impulsivity, neurodevelopmental, and externalizing symptoms in females [64].

Finally, by demonstrating that PRS derived from a GWAS meta-analysis of clinically diagnosed and population trait ADHD are also linked to a wide range of childhood psychopathology problems in the general population, this study further highlights the utility of recently developed multivariate GWAS methods [65–67]. Possibilities for joint analysis of GWAS data across psychiatric conditions, and across clinical and population samples, are important not only to boost power, but also to identify genetic factorswhich influence broader psychopathology dimensions [57].

## Limitations

Results from this study must be interpreted in the context of the study limitations. First, we relied on parent ratings of childhood psychiatric symptoms, which may inflate cross-trait covariance and possibly lead to overestimation of the general psychopathology factor [62]. Although we cannot exclude this possibility, loadings and variance explained by the general psychophatology factor in this study was generally in line with previous findings using multiple raters [62] or register-based clinical diagnoses [33]. Second, the current study is cross-sectional. Although there is strong evidence for genetic stability in ADHD [23, 68], the phenotypic expression of psychiatric symptoms changes across development, meaning that the pattern and strength of ADHD PRS associations may also differ with age [23, 41]. Third, families who participated in CATSS generally had lower rates of psychiatric disorder compared with non-responders [36]. Further, the current study sample had significantly lower rates of ADHD compared with CATSS participants who did not provide genotype data (supplementary Table S2). It is therefore likely that children with higher levels of psychopathology and genetic load for ADHD were underrepresented in this study, which may have attenuated the estimated associations. Fourth, the trait variance explained by PRS is generally small; in the recent clinical ADHD GWAS, ADHD PRS accounted for only ~ 5.5% of the variance in case–control ADHD status [8]. Genetic cross-disorder overlap identified in secondary PRS studies is typically even smaller (< 1% variance explained), and this study is no exception. Thus, our findings very likely do not reflect the total genetic overlap between ADHD and related childhood psychopathology. As the predictive utility of PRS are largely a function of the power of GWAS discovery samples from which the scores were derived, association testing with PRS and comparison of results across PRS studies will likely improve as GWAS discovery sample sizes increase [40, 53]. Such developments have been seen for schizophrenia and in other areas of medicine [53, 69]. Finally, PRS analyses do not distinguish the specific genetic loci driving the observed PRS-trait associations. We were therefore unable to separate genetic variants underpinning the association with specific hyperactivity/impulsivity, from those associated with a general liability towards childhood psychopathology.

## Conclusion

Results from this study indicate that genetic risk variants associated with ADHD and captured by PRS also influence a more a general genetic liability toward broad childhood psychopathology. Beyond contributing to shared genetic liability, ADHD PRS also seem to capture genetic risk with stronger and/or unique effects on hyperactivity/impulsivity. Our findings emphasize the utility of adopting a more dimensional, multivariate framework, and the need to account for the inter-related nature of psychiatric conditions when studying the genetic architecture of childhood psychopathology [57].

## Electronic supplementary material

Supplementary Materials
