# Supplementary material for: The contribution of common genetic risk variants for ADHD to a general factor of childhood psychopathology
Source: Mol Psychiatry. 2018 Jun 22;25(8):1809–21. doi: 10.1038/s41380-018-0109-2 (PMC6169728; doi:10.1038/s41380-018-0109-2)
Supplement: Supplementary file 1 — Supplementary Materials [file 41380_2018_109_MOESM1_ESM.docx]

**Supplementary Materials**

**The contribution of common genetic risk variants for ADHD to a general factor of childhood psychopathology**

**Supplementary Note 1.**

**Genotyping, quality control, imputation, and principal components analyses of genetic data in the Child and Adolescent Twin Study in Sweden (CATSS)**

A total of 11,551 CATSS twins were genotyped using the Illumina Infinium PsychArray-24 BeadChip. Prior to analysis, stringent quality control (QC) procedures were performed on the genotyped markers and individuals using standardized procedures. See Supplementary Figure S1 for a descriptions of the QC protocol. After QC, 561,187 genotyped SNPs and 11,081 samples were retained. Genotypes for another 2,495 MZ twins were imputed from their genotyped co-twin. Genotype imputation was performed in Minimac3^1^ for 13,576 CATSS samples on autosomes using 1000-Genomes data (Phase 3, Version.5) as the reference panel.^2^

To account for population stratification, principal components (PCs) were derived in CATSS using PLINK after LD-pruning and removing genotyped SNPs located in long-range LD regions. We calculated PCs on unrelated individuals and then projected the PCs onto the relatives. The top 20 PCs were extracted and the first 6 retained as covariates, as visual inspection of PC plots suggested little population stratification beyond 6 PCs in this relatively homogenous sample.

**Figure S1. Flow chart of quality control protocol and population selection in CATSS**

Total CATSS sample
 (n~30,000)

**QC SNP and sample exclusions**

- Markers call rate < 98%
- Markers with >10% discordant genotypes among 37 cross-batch duplicate samples
- Markers with more than one discordant genotype among 84 pairs of MZ twins
- Hardy-Weinberg Equilibrium < 1e-6)
- Markers with large allele frequency differences from 1000 Genome European samples and mean GenCall scores<0.5
- Markers associated with more than 1 genotyping batch (at p<5e-8)
- Markers on Y-chromosome or mitochondrial markers with poor variant calling
- Samples with call rate < 98%
- Samples with unusual heterozygosity (autosomal inbreeding coefficient F outside +/- 0.2)
- Samples with evidence of possible sample contamination (> 6 standard deviations from the mean of average sample relatedness in a random set of 1000 samples)
- Samples with sex violation (male with X-chr F < 0.5 or female with X-chr F >= 0.5)
- Non-European ancestral outliers (> 6 standard deviations from the mean values of the first two principal components in 1000-Genomes European populations)

Genotyped CATSS twins

n=11,551

Post QC samples retained

n=11,081

2,495 MZ twin genotypes

imputed from co-twin

n=13,576

n SNPs= 561,187

Total n with

genotype and phenotype data

n=6603 A-TAC subsample

n=6854 SMFQ/SCARED subsample

**Phenotype exclusions**

- Missing data on trait items, n=12
- Down Syndrome/Chromosomal abnormalities, n=9
- Cerebral Palsy, n=65
- Brain Damage/Injury, n=33

**Table S1. Characteristics of CATSS participants born 1992–2005** **according to availability of genetic data**

|  |  | Analysis sample N=13,457 | |  | No  genetic data  N=11,965 | |  | |  |  |
| --- | --- | --- | --- | --- | --- | --- | --- | --- | --- | --- |
|  |  |  |  |  |  |  |  |  | |  |
| Sex |  | N | % |  | N | % | OR | 95% CI | | *p* |
|  | Male = 0 | 6709 | 50% |  | 6211 | 52% | 1.09 | 1.03-1.15 | | <.001 |
|  | Female =1 | 6748 | 50% |  | 5754 | 48% |  |  | |  |
|  |  |  |  |  |  |  |  |  | |  |
| Parental education | |  |  |  |  |  |  |  | |  |
|  | Low | 227 | 2% |  | 370 | 3% | 1.20 | 1.13-1.28 | | <.0001 |
|  | Medium | 5567 | 41% |  | 5230 | 44% |  |  | |  |
|  | High | 7524 | 56% |  | 6160 | 51% |  |  | |  |
|  |  |  |  |  |  |  |  |  | |  |
| ADHD ICD diagnosis | |  |  |  |  |  |  |  | |  |
|  | Cases | 442 | 3% |  | 562 | 5% | 0.69 | 0.60-0.80 | | <.0001 |
|  |  |  |  |  |  |  |  |  | |  |
| ADHD sum score | |  |  |  |  |  |  |  | |  |
|  | N total | 13,449 | 100% |  | 11,878 | 99% | 0.96 | 0.96-0.97 | | <.0001 |
|  |  |  |  |  |  |  |  |  | |  |
|  |  |  |  |  |  |  |  |  | |  |

Note: Odds ratios (OR) were estimated using logistic regression in STATA (StataCorp. 2017. Stata Statistical Software: Release 15), with a cluster robust sandwich estimator to remove distributional assumptions and correct confidence interval (CI) for the clustered nature of twin data. OR represents the association between sample characteristic and participation with genetic data in CATSS. Analysis sample refers to CATSS participants with genetic and phenotypic data who contributed to the current study (451 samples with genetic data were excluded based on failed QC, exclusion criteria, or missing data on relevant variables for the current study). No genetic data refers to participants who contributed phenotypic data via the telephone interview, but did not submit saliva samples for DNA extraction. Parental education refers to mothers and/or fathers highest attained education according to self-report and were coded as follows: Low=compulsory education, usually 9 years; Medium=upper secondary school, vocational training or community college; High=university education. ADHD International Classification of Diseases and Related Health Problems (ICD) diagnosis are based on life-time discharge diagnoses in the Swedish National Patient Register coded according to ICD-10. ADHD sum score reflects the total sum score of parent-ratings on the Autism-Tics, ADHD, and Other Comorbidities inventory (A-TAC) inattentive and hyperactive/impulsive scales.

**Supplementary Note 2.**

**Description of the discovery GWAS meta-analysis used to derive ADHD polygenic risk scores in CATSS**

Polygenic risk scores (PRS) were generated in CATSS based on summary statistics from a meta-analysis of the two largest GWAS of ADHD available to date. The GWAS of clinically diagnosed ADHD included 20,183 cases and 35,191 controls and was conducted by The Lundbeck Foundation Initiative for Integrative Psychiatric Research (iPSYCH)^3^ and the Psychiatric Genomics Consortium (PGC) ADHD working group.^4^ The GWAS of ADHD symptoms included 17,666 children and was conducted by the EArly Genetics and Lifecourse Epidemiology Consortium (EAGLE).^5^ Meta-analysis of the two ADHD GWAS was conducted using a new method, relying on modified sample size-based weights to account for the respective heritabilities, genetic correlation, and measurement scale of each GWAS; See supplementary materials in Demontis et al (2017) for details.^4^ The genetic correlation between the two GWAS was 0.94, with no evidence of genome-wide significant heterogeneity across the samples.^4^ These results suggest that this ADHD GWAS meta-analysis should provide the most powerful discovery sample available to derive ADHD PRS. Although ADHD GWAS results from 23andMe were also available for analyses to the iPSYCH/PGC ADHD working group, these were not included in the ADHD discovery meta-analyses due to lower genetic correlation and evidence of genome-wide significant heterogeneity with clinical ADHD.^4^

**Table S2. ICD diagnoses and A-TAC DSM-based diagnoses in the CATSS analysis sample (N=** **13457)**

| Diagnosis | ICD code | ICD diagnoses (N) | % | DSM  diagnoses (N) | % |
| --- | --- | --- | --- | --- | --- |
| ADHD | F90 | 442 | 3.28% | 600 | 4.46% |
| ASD | F84 | 180 | 1.34% | 146 | 1.08% |
| Scholastic disorders | F81 | 66 | 0.49% | na | na |
| Language disorders | F80 | 116 | 0.86% | na | na |
| Intellectual disability | F70-F79 | 87 | 0.65% | na | na |
| ODD | F91.3 | 13 | 0.10% | 168 | 1.25% |
| CD | F91 | 29 | 0.22% | 22 | 0.16% |
| Depression | F32-F33 | 221 | 1.64% | na | na |
| Anxiety | F40-F41,F93 | 265 | 1.97% | na | na |

Note: International Classification of Diseases and Related Health Problems (ICD) diagnoses are based on life-time discharge diagnoses in the Swedish National Patient Register coded according to ICD-10. DSM-based diagnoses were derived from parent-ratings on the Autism-Tics, ADHD, and Other Comorbidities inventory (A-TAC), and were only available for conditions where the symptoms items assessed closely mapped onto symptom criteria in the Diagnostic and Statistical Manual of Mental Disorders (DSM) IV revision.

**Supplementary Note 3.**

**Brief description of sensitivity analyses**

Increasingly restricted sample size and exclusion of ADHD cases in the sensitivity analyses resulted in estimation problems of the polychoric correlations, particularly for combinations of items that were rarely endorsed. Therefore, all symptom items were dichotomized (0/1) by collapsing the response categories "yes, to some extent" and "yes" and tetrachoric correlations were estimated instead. All sensitivity analyses presented in Table S3 were run on dichotomized symptom items.

In the models exploring sex differences in the association between ADHD PRS and the latent factors, we fitted a multiple-group model in which loadings and thresholds were constrained to be the same across sex, but factor means and regression weights were allowed to vary across sex.^6^ This way, the outcomes (i.e., the latent factors in the general factor model) were the same in both sexes (i.e., invariant), enabling comparison of PRS associations across males and females.

**Supplementary references**

1. Das, S. *et al.* Next-generation genotype imputation service and methods. *Nat Genet* **48**, 1284-1287 (2016).

2. The Genomes Project, C. A global reference for human genetic variation. *Nature* **526**, 68-74 (2015).

3. Pedersen, C.B. *et al.* The iPSYCH2012 case-cohort sample: new directions for unravelling genetic and environmental architectures of severe mental disorders. *Mol Psychiatry* (2017).

4. Demontis, D. *et al.* Discovery Of The First Genome-Wide Significant Risk Loci For ADHD. *bioRxiv* (2017).

5. Middeldorp, C.M. *et al.* A Genome-Wide Association Meta-Analysis of Attention-Deficit/Hyperactivity Disorder Symptoms in Population-Based Pediatric Cohorts. *J Am Acad Child Adolesc Psychiatry* **55**, 896-905.e6 (2016).

6. Muthén, B. & Asparouhov, T. Latent variable analysis with categorical outcomes: Multiple-group and growth modeling in Mplus. *Mplus web notes* **4**, 1-22 (2002).

**Table S3. Sensitivity analyses of the association between ADHD PRS and latent factors in the general factor model**

|  | **A-TAC subsample** | | | |  | **SCARED/MFQ Subsample** | | | |
| --- | --- | --- | --- | --- | --- | --- | --- | --- | --- |
| Latent factor | Beta | S.E | *p* | R^2^ |  | Beta | S.E | *p* | R^2^ |
| **Model S1. ADHD cases excluded** | | | | | | | | | |
| GP | 0.06 | 0.02 | **<.0001** | **0.36%** |  | 0.05 | 0.02 | **0.005** | **0.25%** |
| IA | 0.01 | 0.02 | 0.870 | 0.01% |  | 0.02 | 0.02 | 0.420 | 0.04% |
| H/I | 0.04 | 0.02 | **0.043** | **0.16%** |  | 0.09 | 0.02 | **<.0001** | **0.81%** |
| ASD | -0.02 | 0.02 | 0.508 | 0.04% |  | 0.01 | 0.02 | 0.695 | 0.01% |
| LD | 0.01 | 0.03 | 0.695 | 0.01% |  | 0.01 | 0.03 | 0.845 | 0.01% |
| ODD | -0.01 | 0.03 | 0.880 | 0.01% |  | 0.05 | 0.02 | **0.024** | **0.25%** |
| CD | 0.02 | 0.04 | 0.673 | 0.04% |  | 0.08 | 0.03 | **0.026** | **0.64%** |
| DEP | -0.02 | 0.02 | 0.378 | 0.04% |  | 0.04 | 0.02 | 0.101 | 0.16% |
| ANX | -0.01 | 0.03 | 0.797 | 0.01% |  | - | - | - |  |
| PD | - | - | - | - |  | 0.04 | 0.03 | 0.196 | 0.16% |
| GAD | - | - | - | - |  | -0.01 | 0.02 | 0.967 | 0.01% |
| SAD | - | - | - | - |  | -0.03 | 0.02 | 0.170 | 0.09% |
| SA | - | - | - | - |  | -0.05 | 0.03 | 0.110 | 0.25% |
| **SP** | **-** | **-** | **-** | **-** |  | **-0.05** | **0.02** | **0.011** | **0.25%** |
| **Model S2. Including only 1 MZ twin per complete twin pair** | | | | | | | | | |
| GP | 0.01 | 0.02 | **<.0001** | **0.01%** |  | **0.06** | **0.02** | **0.004** | **0.36%** |
| IA | 0.00 | 0.03 | 0.893 | 0.00% |  | 0.01 | 0.02 | 0.634 | 0.01% |
| H/I | 0.05 | 0.02 | **0.036** | **0.25%** |  | **0.09** | **0.02** | **<.0001** | **0.81%** |
| ASD | 0.01 | 0.03 | 0.599 | 0.01% |  | -0.01 | 0.02 | 0.797 | 0.01% |
| LD | -0.01 | 0.03 | 0.733 | 0.01% |  | -0.01 | 0.03 | 0.851 | 0.01% |
| ODD | 0.00 | 0.03 | 0.993 | 0.00% |  | 0.04 | 0.02 | 0.080 | 0.16% |
| CD | 0.04 | 0.04 | 0.359 | 0.16% |  | 0.07 | 0.04 | 0.075 | 0.49% |
| DEP | 0.01 | 0.03 | 0.717 | 0.01% |  | 0.02 | 0.02 | 0.321 | 0.04% |
| ANX | -0.01 | 0.02 | 0.648 | 0.01% |  | - | - | - | - |
| PD | - | - | - | - |  | 0.04 | 0.03 | 0.136 | 0.16% |
| GAD | - | - | - | - |  | 0.00 | 0.02 | 0.887 | 0.00% |
| SAD | - | - | - | - |  | -0.01 | 0.02 | 0.549 | 0.01% |
| SA | - | - | - | - |  | -0.03 | 0.03 | 0.304 | 0.09% |
| **SP** | **-** | **-** | **-** | **-** |  | **-0.04** | **0.02** | **0.022** | **0.16%** |
| **Males** | | | | | | | | | |
| GP | 0.12 | 0.02 | **<.0001** | **1.44%** |  | 0.10 | 0.02 | **<.0001** | **1.00%** |
| IA | -0.01 | 0.03 | 0.913 | 0.01% |  | 0.04 | 0.03 | 0.147 | 0.16% |
| H/I | 0.09 | 0.03 | **<.001** | **0.81%** |  | 0.09 | 0.03 | **0.002** | **0.81%** |
| ASD | 0.01 | 0.03 | 0.970 | 0.01% |  | 0.01 | 0.03 | 0.676 | 0.01% |
| LD | -0.02 | 0.04 | 0.568 | 0.04% |  | -0.01 | 0.04 | 0.836 | 0.01% |
| ODD | 0.01 | 0.03 | 0.740 | 0.01% |  | 0.03 | 0.03 | 0.321 | 0.09% |
| CD | 0.04 | 0.05 | 0.498 | 0.16% |  | 0.09 | 0.05 | 0.076 | 0.81% |
| DEP | -0.02 | 0.03 | 0.469 | 0.04% |  | 0.01 | 0.03 | 0.635 | 0.01% |
| ANX | 0.01 | 0.04 | 0.726 | 0.01% |  | - | - | - | - |
| PD | - | - | - | - |  | 0.03 | 0.04 | 0.756 | 0.09% |
| GAD | - | - | - | - |  | -0.01 | 0.03 | 0.921 | 0.01% |
| SAD | - | - | - | - |  | -0.03 | 0.03 | 0.281 | 0.09% |
| SA | - | - | - | - |  | -0.02 | 0.04 | 0.700 | 0.04% |
| SP | - | - | - | - |  | -0.06 | 0.02 | 0.020 | 0.36% |
| **Females** | | | | | | | | | |
| GP | 0.06 | 0.03 | **0.027** | **0.36%** |  | 0.09 | 0.03 | **<.001** | **0.81%** |
| IA | -0.00 | 0.03 | 0.985 | 0.00% |  | 0.00 | 0.03 | 0.913 | 0.00% |
| H/I | 0.01 | 0.03 | 0.650 | 0.03% |  | 0.10 | 0.03 | <.001 | **1.00%** |
| ASD | 0.01 | 0.04 | 0.831 | 0.01% |  | 0.07 | 0.03 | **0.041** | **0.49%** |
| LD | 0.03 | 0.04 | 0.498 | 0.09% |  | -0.05 | 0.04 | 0.243 | 0.25% |
| ODD | 0.00 | 0.04 | 0.938 | 0.00% |  | 0.05 | 0.03 | 0.082 | 0.25% |
| CD | 0.03 | 0.06 | 0.666 | 0.24% |  | 0.06 | 0.04 | 0.142 | 0.36% |
| DEP | 0.01 | 0.03 | 0.910 | 0.00% |  | 0.03 | 0.03 | 0.288 | 0.09% |
| ANX | -0.01 | 0.04 | 0.744 | 0.01% |  | - | - | - | - |
| PD | - | - | - | - |  | 0.02 | 0.04 | 0.569 | 0.04% |
| GAD | - | - | - | - |  | -0.02 | 0.03 | 0.543 | 0.04% |
| SAD | - | - | - | - |  | -0.03 | 0.03 | 0.203 | 0.09% |
| SA | - | - | - | - |  | -0.08 | 0.04 | **0.023** | **0.64%** |
| SP | - | - | - | - |  | -0.04 | 0.02 | 0.074 | 0.16% |

**Model S1.** Excluding ADHD cases based on ICD diagnosis of ADHD obtained from the Swedish National Patient Register or a DSM-based diagnoses derived from parent-ratings on the inattention and hyperactivity/impulsivity scales in A-TAC. Total N cases excluded: A-TAC subsample N=431, SMFQ/SCARED subsample N=433.

**Model S2**. Including only 1 randomly selected MZ twin per complete MZ twin pair, every twin from MZ non-complete pairs and all DZ twins (A-TAC subsample N=5283, SMFQ/SCARED subsample N=5288).

**N for sex-specific PRS associations:**

Males: A-TAC subsample N=3331, SMFQ/SCARED subsample N=3378.

Females: A-TAC subsample N= 3272, SMFQ/SCARED subsample N=3476.

**Note:** All models were adjusted for age and six principal components. Model S1-S2 were further adjusted for sex. Reported betas are standardized. R^2^, represent % variance explained (beta^2^). GP, general psychopathology factor. IA, inattention. H/I, hyperactivity/impulsivity. ASD, autism spectrum disorder. LD, learning difficulties. ODD, oppositional defiant disorder. CD, conduct disorder. DEP, depression. ANX, anxiety. PD, panic disorder. GAD, generalized anxiety disorder. SAD, separation anxiety disorder. SA, school anxiety. SP, social phobia.

**
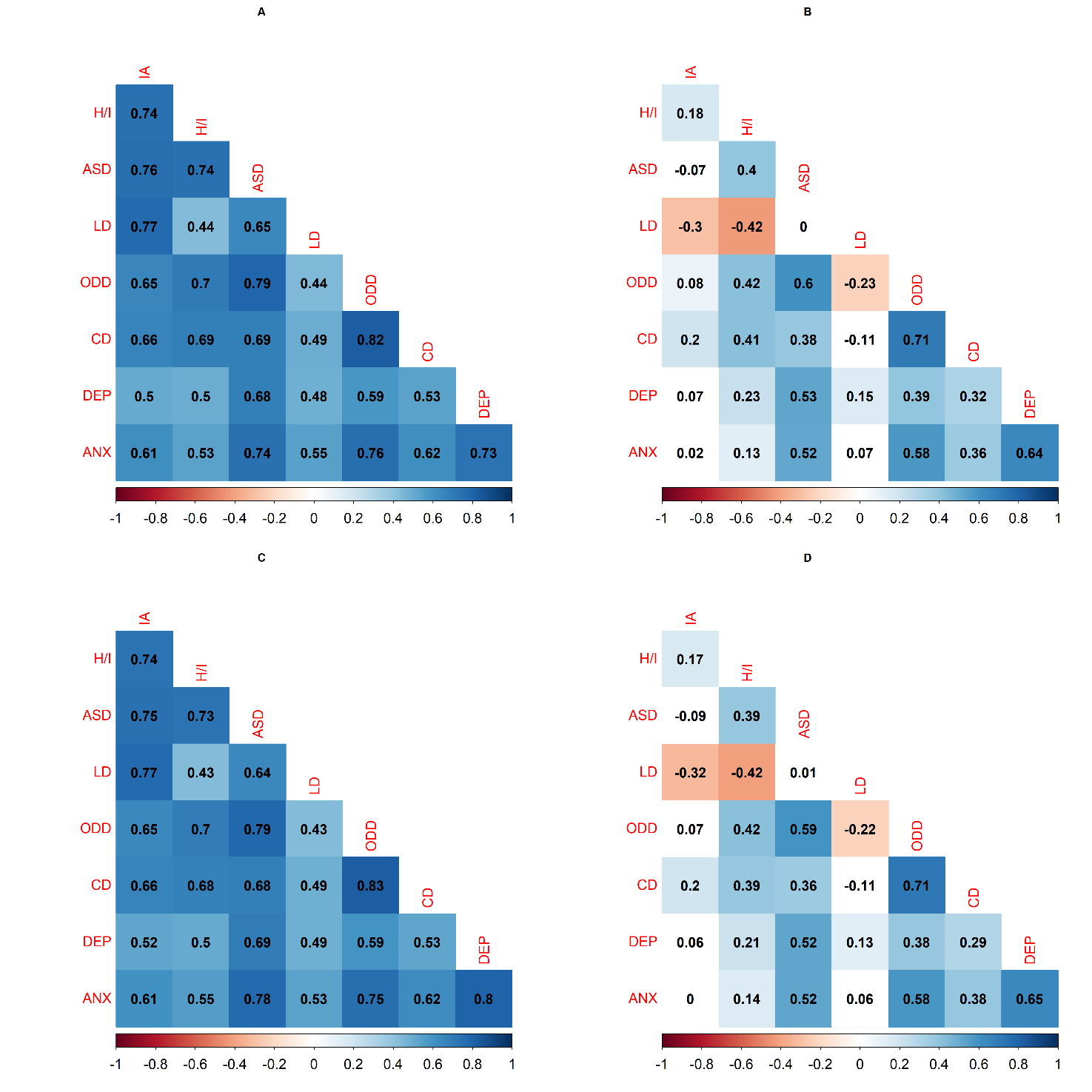
**

**Figure S2.** Correlations across latent traits factors from the correlated factors model and the general factor model before and after regression of ADHD PRS, sex, age and principal components in the A-TAC subsample

Note: Panel A, correlated factors model prior to regression. Panel B, general factor model prior to regression. Panel C, full SEM correlated factors model including regressions of latent factors on ADHD PRS, sex, age and 6 principal components. Panel D, full SEM general factor model including regressions of latent factors on ADHD PRS, sex, age and 6 principal components. IA, inattention factor. H/I, hyperactivity/impulsivity factor. ASD, autism spectrum disorder factor. LD, learning difficulties factor. ODD, oppositional defiant disorder factor. CD, conduct disorder factor. DEP, depression factor. ANX, anxiety factor.

**
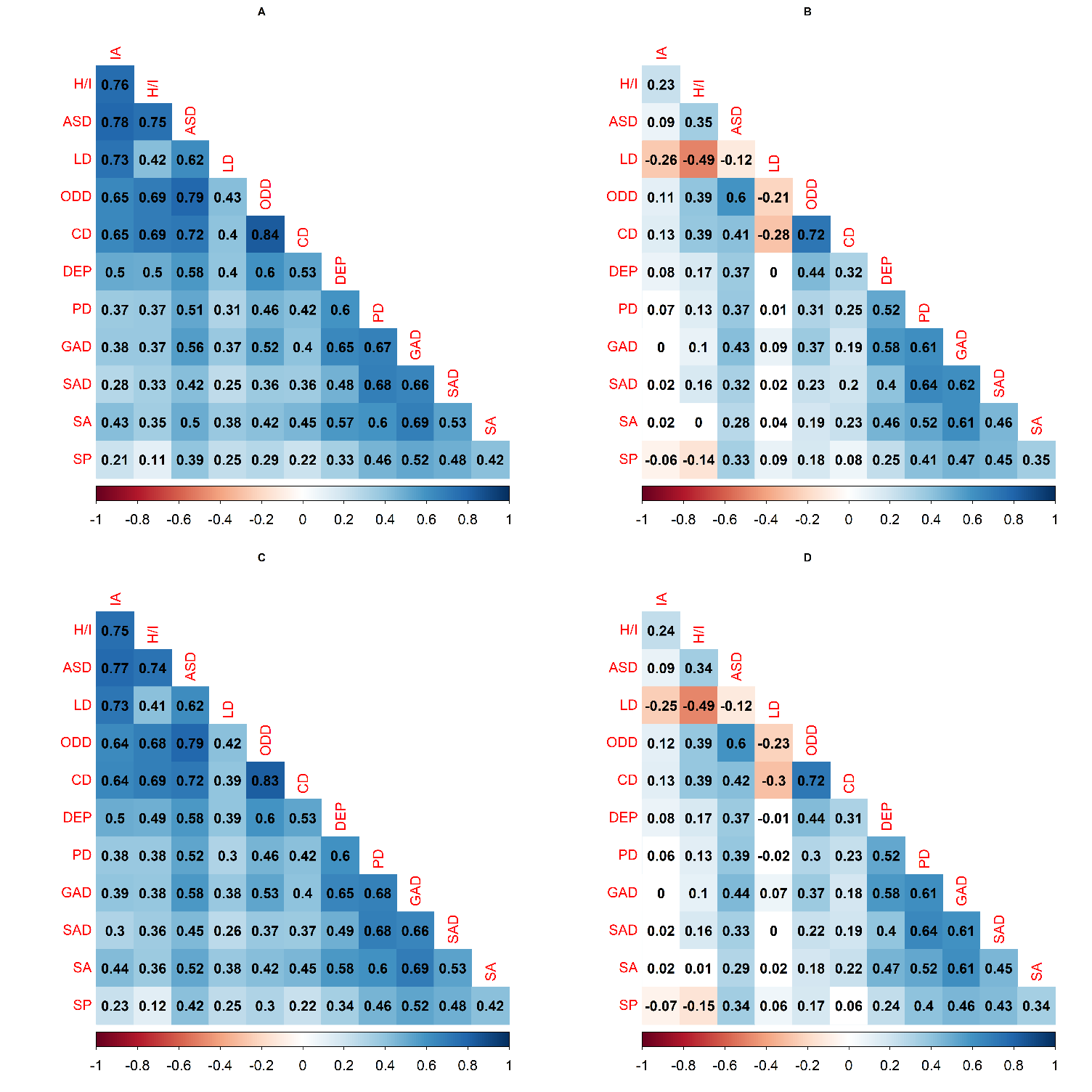
**

**Figure S3.** Correlations across latent traits factors from the correlated factors model and the general factor model before and after regression of ADHD PRS, sex, age and principal components in the SCARED-SMFQ subsample

Note: Panel A, correlated factors model prior to regression. Panel B, general factor model prior to regression. Panel C, full SEM correlated factors model including regressions of latent factors on ADHD PRS, sex, age and 6 principal components. Panel D, full SEM general factor model including regressions of latent factors on ADHD PRS, sex, age and 6 principal components. IA, inattention factor. H/I, hyperactivity/impulsivity factor. ASD, autism spectrum disorder factor. LD, learning difficulties factor. ODD, oppositional defiant disorder factor. CD, conduct disorder factor. DEP, depression factor. PD, panic disorder factor. GAD, generalized anxiety disorder factor. SAD, separation anxiety disorder factor. SA, school anxiety factor. SP, social phobia factor.


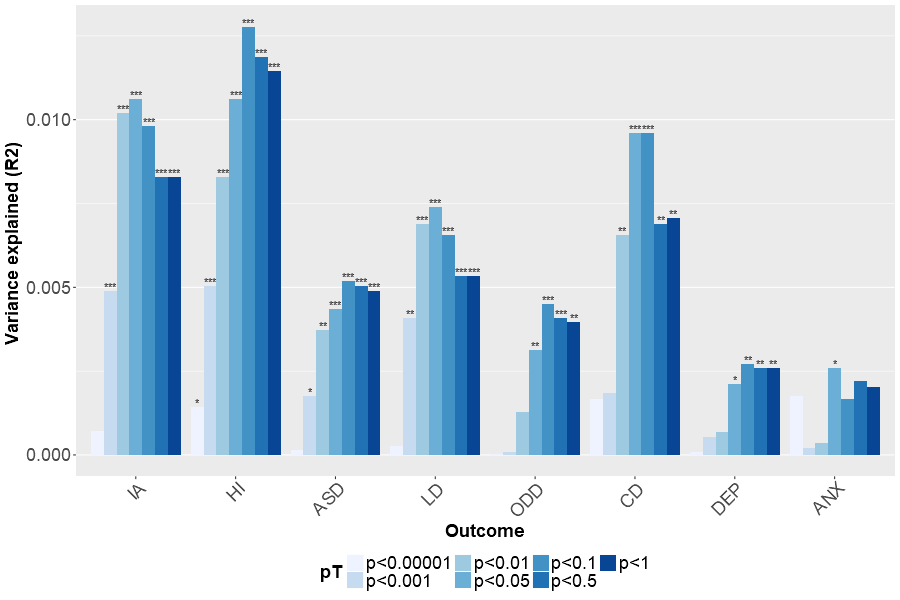


**A**

**B**


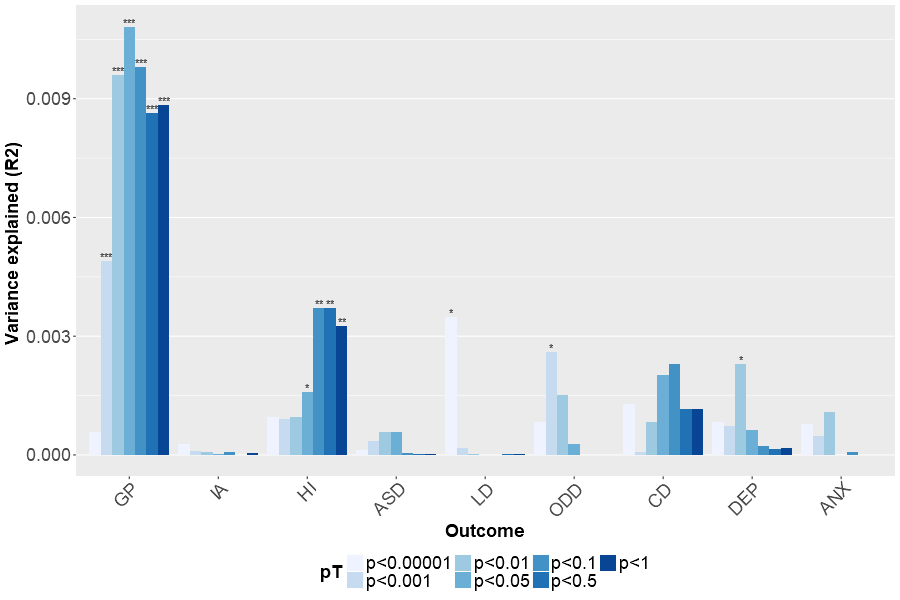


**Figure S4.** Variance explained by ADHD PRS in latent factors from the correlated factors model (A) and the general factor model (B) across PRS *p*-value thresholds in the A-TAC subsample.

Note: R^2,^ represent % variance explained (beta^2^). GP, general psychopathology factor. IA, inattention factor. H/I, hyperactivity/impulsivity. ASD, autism spectrum disorder. LD, learning difficulties. ODD, oppositional defiant disorder. CD, conduct disorder. DEP, depression. ANX, anxiety.

**
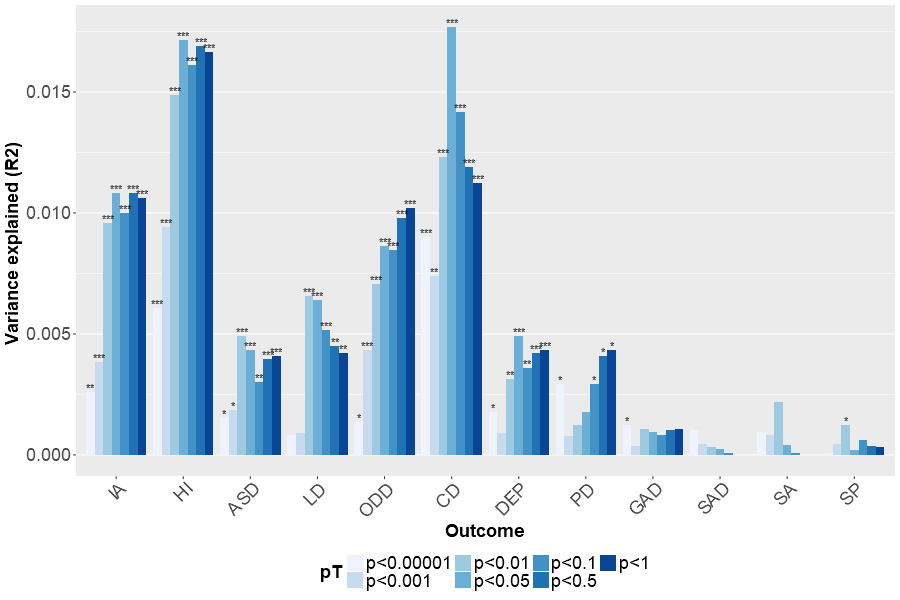
**

**A**

**B**

**
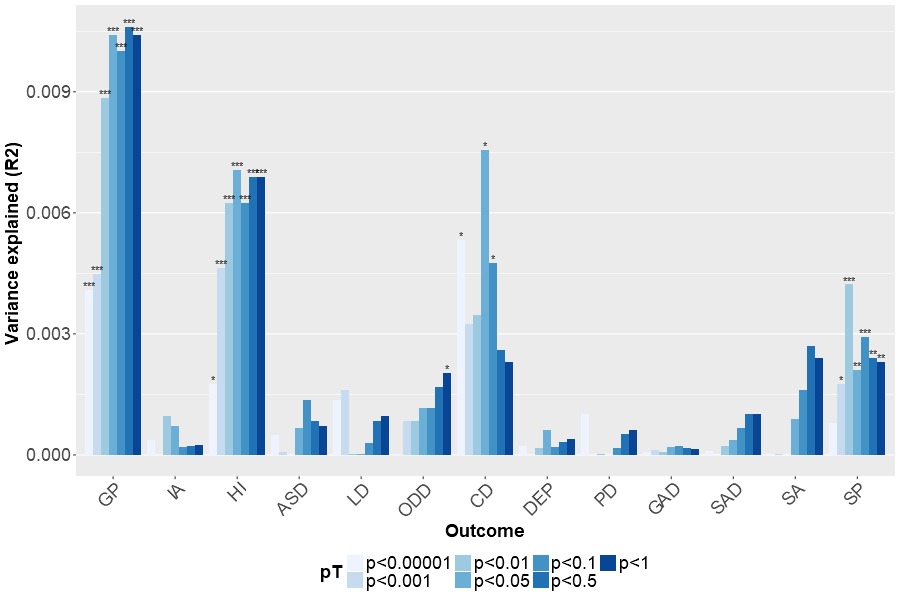
**

**Figure S5.** Variance explained by ADHD PRS in latent factors from the correlated factor model (A) and the general factor model (B) across PRS *p*-value thresholds in the SMFQ/SCARED subsample.

Note: R^2,^ represent % variance explained (beta^2^). GP, general psychopathology factor. IA, inattention factor. H/I, hyperactivity/impulsivity factor. ASD, autism spectrum disorder factor. LD, learning difficulties factor. ODD, oppositional defiant disorder factor. CD, conduct disorder factor. DEP, depression factor. PD, panic disorder factor. GAD, generalized anxiety disorder factor. SAD, separation anxiety disorder factor. SA, school anxiety factor. SP, social phobia factor.
